# Feeding Your Himalayan Expedition: Nutritional Signatures and Body Composition Adaptations of Trekkers and Porters

**DOI:** 10.3390/nu13020460

**Published:** 2021-01-30

**Authors:** Danilo Bondi, Anna Maria Aloisi, Tiziana Pietrangelo, Raffaela Piccinelli, Cinzia Le Donne, Tereza Jandova, Stefano Pieretti, Mattia Taraborrelli, Carmen Santangelo, Bruna Lattanzi, Vittore Verratti

**Affiliations:** 1 Department of Neuroscience, Imaging and Clinical Sciences, University “G. d’Annunzio” of Chieti—Pescara, 66100 Chieti, Italy; danilo.bondi@unich.it (D.B.); matti.taraborrelli@gmail.com (M.T.); carmensantangelo17@gmail.com (C.S.); 2 Department of Medicine, Surgery and Neuroscience, University of Siena, 53100 Siena, Italy; annamaria.aloisi@unisi.it; 3 Council for Agricultural Research and Economics, Research Centre for Food and Nutrition, 00178 Rome, Italy; raffaela.piccinelli@crea.gov.it (R.P.); cinzia.ledonne@crea.gov.it (C.L.D.); 4 Faculty of Physical Education and Sport, Charles University, 116 36 Prague, Czech Republic; te.jandova@hotmail.com; 5 National Center for Drug Research and Evaluation, Istituto Superiore di Sanità, 00161 Rome, Italy; stefano.pieretti@iss.it; 6 Faculty of Science and Technology, Free University of Bozen, 39100 Bolzano, Italy; bruna.lattanzi@hotmail.it; 7 Department of Psychological, Health and Territorial Sciences, University “G. d’Annunzio” of Chieti—Pescara, 66100 Chieti, Italy; vittore.verratti@unich.it

**Keywords:** food diary, altitude, trekking, bioimpedance, anthropometrics, muscle ultrasound, micronutrients, macronutrients, dietary habit

## Abstract

High-altitude exposure leads to many physiological challenges, such as weight loss and dehydration. However, little attention has been posed to the role of nutrition and ethnic differences. Aiming to fulfill this gap, five Italian trekkers and seven Nepalese porters, all males, recorded their diet in diaries during a Himalayan expedition (19 days), and the average daily intake of micro and macro-nutrients were calculated. Bioimpedance analysis was performed five times during the trek; muscle ultrasound was performed before and after the expedition, only for the Italians. The Nepalese group consumed a lot of rice and only Italians consumed cheese. Water intake was slightly over 3000 g/d for both groups. Nepalese diet had a higher density of dietary fibre and lower density of riboflavin, vitamins A, K, and B12. Intake of calcium was lower than recommended levels. Body mass index, waist circumference, fat-free mass, and total body water decreased in both groups, whereas resistance (Rz) increased. Italians reactance (Xc) increased at day 9, whereas that of Nepalese occurred at days 5, 9, and 16. The cross-sectional area of the *Vastus lateralis* was reduced after the expedition. Specific nutritional and food-related risk factors guidance is needed for diverse expedition groups. Loss of muscle mass and balance of fluids both deserve a particular focus as concerns altitude expeditions.

## 1. Introduction

Harsh environments elicit a plethora of specific physiological adaptations, alter body composition, performance and require special nutritional requirements [1]. As altitude travelling is increasing, novel insights about the specific adaptations and requirements are needed. In particular, beyond a massive literature on the topic [2,3], studies conducted with an ecological design are missing. The specific physiological signature of altitude population for dealing with acute and chronic hypobaric hypoxia exposure have been studied often in terms of metabolic and genetic bases [4,5]. However, little attention has been posed to the role of nutrition, which may help to unravel some mechanisms underlying altitude adaptations [6]. Moreover, the focus has been posed to highland dwellers and to lowland trekkers and climbers. Therefore, there is a lack of evidence on those who are generally lowland dwellers working at high altitudes, such as porters, who logistically support expeditions and are prone to high altitude illnesses [7].

Many authors in recent decades have highlighted the effects of high altitude on nutritional status, metabolism and body composition [8,9,10,11,12,13,14]. Based on these literature reviews, it can be said that there is a shred of evidence that altitude exposure leads to many physiological challenges such as weight loss—in particular of fat free mass [15] and decreased liquid intake—caused by changes of diet and habits and to a metabolic effect elicited by hypobaric hypoxia. The physiology behind weight loss at altitude is constituted by several factors, from an imbalance between intake and expenditure to loss of appetite, changes in body composition and dehydration [15]. Muscular distress provoked by a physical effort at high altitude affects body composition and activates pro-inflammatory pathways [16]. Distress in fluid balance is a typical response to high-altitude exposure, with an altered micturition habit and a loss of body fluids [1,17].

To evaluate body composition, bioimpedance analysis (BIA) devices are more practical and portable compared to the most accurate equipment, such as dual-energy X-ray absorptiometry. Avoiding the typical concerns associated with predictive equations, the use of bioimpedance vector analysis (BIVA) in sport and exercise fields has grown for its reliability in monitoring the changes in body fluids. The vector considers both resistance (Rz), which arises from extra-cellular and intracellular water, and capacitive reactance (Xc), which represents the cell membrane’s capacity for capturing electric load and releasing it after a phase delay. As recently reported [18], there has been an increasing interest in the use of BIA in monitoring adaptations induced by exercise and sport. Novel pieces of evidence are emerging to identify the vectors and parameters for athletes [19]. Moreover, body composition changes due to physical stress may be detrimental for performance and health and monitoring fluid variation should help to prescribe adequate fluid intake. Short-term and long-term vector changes should be preferred with respect to tolerance ellipses for comparing athlete status to reference populations [18].

Into the topic of muscular examination, muscle ultrasound has been demonstrated as a useful, non-invasive, feasible and reliable tool across all the ages [20,21]. The body water and muscle mass loss during and after altitude exposure linked to the altered nutritional requirements [1] supports the need for quantitative assessments of intake during the expeditions.

Monitoring the diet in these particular environmental conditions becomes essential for interpreting the physical and metabolic changes at high altitude. Food consumption data reflect what either individuals or groups consume in terms of solid foods, beverages, including drinking water, and supplements. Current tools allow quantifying dietary assessment, with particular insight regarding food grouping, food composition linked to large scale database, and novel methodologies for data processing [22]. Among the various available dietary assessment methods to detect food consumption, the food diary by the household unit of measurement (with quantities reported by household units of measurement and with the aid of an atlas of portions), and the 24 h recall are those that best fit the purposes of nutritional surveillance [23]. The dietary record method seems to provide a higher level of detail since all foods, including beverages and food supplements consumed during the 24 h, are recorded and quantified and described as eaten. A computerized data management system allows processing the collected dietary information (hard copy) into the weight of single raw ingredients and the amounts of nutrients consumed.

We hypothesized that a quantitative monitoring of nutritional intake would have unveiled typical features, and possibly deficiencies, in the dietary habits of travelers vs. porters during a Himalayan expedition, linked to body composition adaptations. Therefore, we aimed to identify any harmful nutritional condition with ecological study design, evaluating Westerner travelers and Himalayan porters during an altitude expedition. In addition, we aimed to extend the understanding of body composition, trying to ascertain how specific parameters change during to a typical altitude expedition, taking into account ethnic origin as a factor.

## 2. Materials and Methods

### 2.1. Study Design and Participants

**Figure 1:**
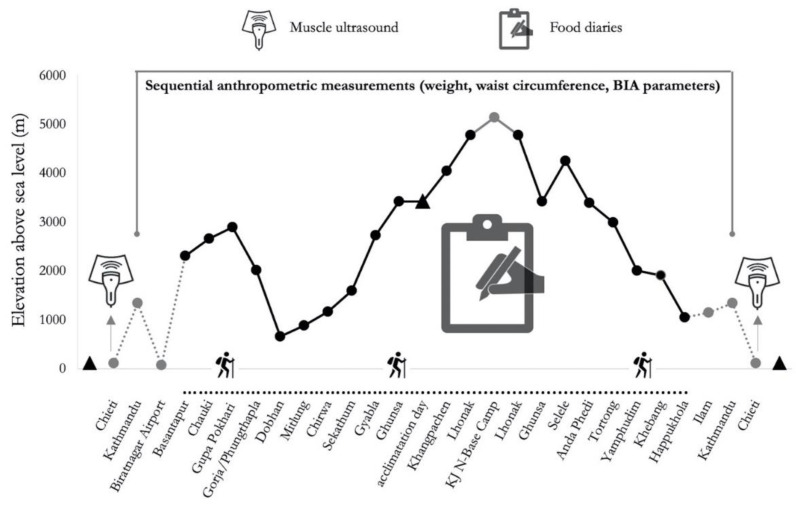
Altimetric scheme of the “Kanchenjunga Exploration and Physiology” project. The continuous line represents the effective trek. The plain data points represent the effective Himalayan trekking expedition. The empty circular data point at Kanchenjunga North Base Camp (KJ N-Base Camp) represents the highest altitude (5143 m) reached by participants throughout the trek.

This study was a part of the research project termed “Kanchenjunga Exploration & Physiology”, belonging to the broader project entitled “Environmentally-modulated metabolic adaptation to hypoxia in altitude natives and sea-level dwellers: from integrative to molecular (proteomics, epigenetics, and ROS) level”. Written informed consent prior to participate in the study was given by all humans. This research was approved by the Ethics Review Board of the Nepal Health Research Council (NHRC, ref. no. 458). All study procedures conformed to the ethical standards set by the Declaration of Helsinki and its subsequent amendments [24]. The expedition was conducted at low (500–2000 m), moderate (2000–3000 m) and high altitude (3000–5500 m) [3], with a traveling plan (Figure 1) to ease acclimation and prevent altitude sickness. The expedition involved two populations, Italian trekkers and Nepalese porters, defined in terms of non-altitude workers and altitude workers, respectively. There were five Italian trekkers (mean age 45.40 ± 16.56 years, mean BMI 25.96 ± 3.61 kg/m^2^), and seven Nepali porters (mean age 29.00 ± 8.33 years, mean BMI 23.79 ± 4.55 kg/m^2^) included in this study. In addition to these men, there was one Italian female trekker, participating in the expedition (age 36 years, BMI 25.07 kg/m^2^). The usual Caucasian trekkers’ dwelling place was at sea level, some of them reported previous high-altitude experiences, albeit none in the last three years. The Nepalese trekkers usually live at low altitude and reported habitual exposure to high altitude, with a working experience of 2–5 high altitude expeditions per year. Participants completed a combined circuit of about 300 Km distance in 19 days, covering a daily average of 6 h walk, along a demanding route with ascent and descent (over 16,000 m of difference in altitude) in the Himalayas, Nepal. The expedition was continuously supervised by a medical doctor with a specific expertise in altitude medicine.

### 2.2. Dietary Assessment

Dietary plans were not defined prior to the expedition because the study was conducted in an ecological field design. After being instructed, participants recorded all food, beverages and supplements ingested in hard-copy diaries structured into seven meals (three principal meals and four snacks), in non-consecutive days during the trek with an interval of 6–7 days. For logistical problems, the Nepalese subjects recorded two diaries (one subject filled in only one), while the Italians filled out three diaries. For every food, beverage, recipe and food supplement eaten they carefully recorded the time and place of consumption, detailing the exact description and quantity of each food, as well as precise recipes, ingredients, conservation and cooking methods. Participants specified the quantities consumed referring to a dedicated picture atlas. All filled food diaries were checked by the expert field worker who later entered data into an ad hoc web-based software database, Food Consumption Database (FOODCONS). This software was also used for data coding and data processing, which allowed to transform each recorded food and recipes into: weight in grams, amount of energy intake (EI), water, macronutrients (proteins, fat, saturated (SFA), monounsaturated (MUFA) and polyunsaturated fatty acids (PUFA), cholesterol, available, complex, and soluble carbohydrates (CHO)), fibers, alcohol, minerals such as calcium (Ca), phosphorus (P), magnesium (Mn), potassium (K), iron (Fe), zinc (Zn) and vitamins such as vitamin C (vit C), thiamine, riboflavin, niacin, vitamin B6 (vit B6), vitamin B12 (vit B12), vitamin D (Vit D), vitamin E (Vit E), vitamin K (Vit K), retinol, vitamin A (expressed in retinol equivalents or REs).

The software allows clustering the intakes by seven meals (three principal meals and four snacks), 16 food categories and 90 food sub-categories. The software FOODCONS and all connected instruments (food composition data bases, the hard-copy food dairies, the picture atlas) were developed by the “Research Center for Food and Nutrition of Council for Agricultural Research and Economics” (CREA—Food and Nutrition) (https://www.crea.gov.it/en/web/alimenti-e-nutrizione) for use in food consumption survey in Italy and ad-hoc adapted for this study.

Energy, macro and some micronutrient intake were finally assessed according to the guidelines of the ‘International Society of Sports Nutrition (ISSN)’ [25] and the ‘Academy of Nutrition and Dietetics (Academy), Dietitians of Canada (DC), and the American College of Sports Medicine (ACSM)’ [26] for specific physical activities. That of the Italian group can be considered as moderate/high levels of intense training (2–3/3–6 h per day of intense exercise performed 5–6 times per week). According to the guidelines and level of physical activity, the energy requirement may approach 40–70 kcal/kg bw/day (2000–7000 kcal/day for a 50–100 kg athlete) [25]; athletes involved in high volume intense training may need to consume 8–10 g/kg bw/day of carbohydrate [25] until 12 g/kg bw/day [26]. Dietary protein intake necessary to support metabolic adaptation, repair, remodelling, and for protein turnover generally ranges from 1.2 to 2.0 g/kg/day up to 2 g/kg bw/day to prevent loss of fat-free mass (FFM) [26] as confirmed by ISSN guidelines which reports a range of 1.7–2.2 g/kg bw/day [25]. Both Institutions claim that the intake of fat by athletes should be in accordance with public health guidelines [25,26], even if up to 50% of daily calories from fat can be safely ingested by athletes during regular high-volume training [25].

The recommended daily allowances of micronutrient intake for the general population can cover the demands of athletes [25,26], except for iron that may be increased up to 70% in specific situations [27]. A multivitamin/mineral supplement in athletes may be appropriate when a diet providing high energy availability from a variety of nutrient-dense foods is not consumed. Finally, according to the ACSM experts daily fluid needs as high as 4 to 5 L with altitude training and competition [26], whereas ISSN encourage individual monitoring of hydration status to determine fluid requirements in athletes [25].

### 2.3. Bioimpedance Analysis

Both Italians and Nepalese were tested for bioelectrical impedance analysis (BIA) five times during the trek. The first and the last measurement were conducted at Kathmandu, one day before and one day after the altitude expedition, respectively. The other measurements were conducted in the 5th, 9th, and 15th day of the trek. BIA uses alternating current (at constant intensity and frequency), injected on the skin by electrodes-patch, which passes through the electrolytic solutions of the extra-cellular liquid and the intra-cellular liquid of all tissues (excluding fat and bone) by generating an impedance-Z vector. The cell membranes and the interfaces of the tissues phase-out the current conduction (phase angle between voltage and current of the vector) generating the capacitive component of impedance-Z, or the reactance-Xc. The vector BIA provides specific values on the conductive tissues, compartment expressed as an Rz-resistance value by the intra and extra-cellular electrolytic solutions, and as an Xc reactance value by the set of cell membranes of the compartment itself. BIA measurements were conducted in a supine position, arms about 30° separated from the trunk and legs apart by about 45°, with the whole-body analysis method. Before the measurement, participants stayed relaxed for 5 min in the supine position. Measurements were conducted in the early morning in a fasting state. In the dorsal part of both hand and feet, after cleaning, two skin electrodes were placed, with a distance between them of about 5 cm. Measurement were conducted with the sole frequency of 50 kHz [28] trough the BIA 101 New Edition device (Akern Srl, Firenze, Italy). If the coefficient of variation of two consecutive measures was <2%, the data of resistance and reactance were recorded, while participants remained stable. Data were analyzed with the Bodygram^TM^ PLUS software Version 1.2.2.8 (Akern Srl) for the real values of Capacitive Reactance (X_C_) and Resistance component of impedance (R_Z_), and the computed value of Phase Angle (PhA), as the arc tangent of the ratio of reactance versus electric resistance [29]. In addition, the following values were estimated by the predictive equation of the used software: basal metabolic rate (BMR), fat mass (FM), fat mass index (FMI), fat free mass (FFM), fat free mass index (FFMI), total body water (TBW), body cellular mass (BCM), skeletal muscle mass (SM), skeletal muscle mass index (SSMI), and the original parameters of Hydragram^TM^ and Nutrigram^TM^. The BIVA method allowed to plot the raw impedance data standardised by the height as a bivariate vector in a nomogram.

### 2.4. Muscle Ultrasound

Italians were tested 10 days before and 7 days after the expedition to examine the muscular adaptations using muscle ultrasound (US) on muscle architecture of Vastus lateralis (VL), including fiber length (FL), pennation angle (PA) and muscle thickness (MT), and *Lumbar multifidus* (LM), including CSA. The logistics did not allow to have the ultrasound scanner during the expedition; therefore, the tests were conducted in Italy and Nepalese porters could not be tested. Muscle ultrasound was performed using a B-mode (Mylab Gamma, EsaoteBiomedica, Genova, Italy) equipped with a 5 cm, 3–11 MHz linear-array probe; all the images were analyzed using the free software Image-J [30] by the same investigator. For VL the protocol by Ticinesi et al. was followed [31]. FL, PA and MT were measured on the right limb of VL at 65% distal distance by placing the probe longitudinally along the long axis. For the images of CSA of VL, the extended field of view software was activated with the probe rotated 90° counterclockwise at the same 65% distal distance [31]. For LM the protocol was based on reported procedures [32,33]. The CSA of resting LM was measured bilaterally at a vertebral level of L5, which was previously palpated and marked by a dermographic pen. Ultrasound was used to confirm and verify the skin marks by placing the probe longitudinally over the lower lumbar spine in the mid-line. After this, the probe was rotated 90° to lie transversely at L5 level in the midline to capture the CSA of LM. During the ultrasound of VL, the volunteer was lying supine on an examination bed with the knee fully extended. During the ultrasound of LM, the volunteer was relaxed in a lying prone position on an examination bed with one or two pillows placed under the hips. Three longitudinal images of VL and three CSA images of VL, and three images of both sides of LM were taken and saved for later analysis [31,32]. 

### 2.5. Statistics

The statistical analysis of nutritional data was carried out using the R-based open-source software Jamovi Version 1.2.5.0 (retrieved from https://www.jamovi.org) and GraphPad Prism Version 6 (GraphPad Software, La Jolla, CA, USA). The assumption check was based on the Shapiro-Wilk and Levene’s tests; consequently, we used Student’s t test, or Welch’s test (with unequal variances), or Mann-Whitney U test (if normality assumption was violated). Anthropometric and BIA parameters were compared using two-way ANOVA and Sidak’s post-hoc test multiple comparison test, calculating partial eta squared (η^2^_p_) and partial omega squared (ω^2^_p_) as measures of effect size [34]. US of Italians collected before and after the expedition, after the assumption checks, were compared with the paired sample t-test. Considering the small sample size, Cohen’s d was adjusted to Cohen’s d_unbiased_ [34].

## 3. Results

The study involved six Italians and seven Nepalese. The only woman who belonged to the Italian group and one Nepalese subject who had recorded only one food dairy were excluded from the analyses. For this reason, the results presented refer to five Italians and six Nepalese. None of the participants suffered from altitude sickness. Participants encountered good weather, with low rainfall and comfortable humidity. Temperature and wind were pleasant across the expedition; regarding the days of measurements, only at day 16 of measurement participants encountered a very cold day, expected due to highest altitude.

### 3.1. Dietary Analysis

Nepalese porters ate mainly noodle soup for breakfast and dal bhat, a common Nepalese recipe with rice, lentils, potatoes and tarkari (curried vegetables), both for lunch and dinner. Nepalese were not used to eating snacks between the main meals. Instead, Italians had a more variable diet, ranging from noodle soup and dal bhat to veg momos (a steamed dumpling stuffed with mixed vegetables) and eggs. The latter ate between the main meals, took supplements (Mg + K or multivitamin) and could eat meat and drink a few alcoholic beverages. A common aspect of the two groups was the frequent consumption of tea throughout the trek, more than once a day. The medical supervisor advocated all the participants for a massive drinking habit in order to prevent the altitude-related dehydration. Italians only took one Diamox (acetazolamide, used to prevent and reduce the symptoms of altitude sickness) per day in three consecutive days before the base camp day.

Table 1 reports the distribution of EI, water and macronutrients intake according to the main meals and snack. In both groups, lunch and dinner are the meals with the highest percentage of energy intake, even if in Nepalese it was greater at the expense of breakfast and snacks percentage which are almost half that of Italians. Water was consumed especially during snacks by the Italians and during lunch and dinner by Nepalese. The highest percentage of proteins were consumed during dinner in both groups. The Nepalese consumed similar percentages of lipids during dinner and lunch, as well as the Italians. Carbohydrates were consumed by Nepalese mostly during lunch and dinner; whereas the Italian group showed a carbohydrates intake distributed over all three main meals with a similar percentage.

The analysis of the food consumption data revealed that participants consumed foods and beverages belonging to 15 food categories and 44 sub-categories. The daily mean amounts in grams of the Italian and Nepalese consumption in terms of food categories and sub-categories are given in Table 2.

“Fish and seafood” is the only food category not consumed by either group. 100% of Italian subjects consumed all other food categories with the exception of “Alcoholic beverages and substitutes” consumed by only three subjects, “Meal substitutes” only by one and “Food supplements” only by three. No Nepalese consumed “Meat, meat products and substitutes”, “Alcoholic beverages and substitutes”, “Meal substitutes”, “Food supplements”, only one subject consumed “Milk, milk products and substitutes”, “Eggs”, and “Miscellaneous” and four subjects “Fruit, fresh and processed”.

For several food categories, the daily mean consumption resulted higher in Italian group, mostly for “Fruit, fresh and processed” (117.3 g/day vs. 20.8 g/day, U = 0, *p* = 0.005, d_unb_ = 4.230), “Milk, milk products and substitutes” (251.4 g/day vs. 5.6 g/day, U = 0, *p* = 0.005, d_unb_ = 2.503), and “Eggs” (108.9 g/day vs. 11.8 g/day, U = 1, *p* = 0.010, d_unb_ = 2.255). Conversely, the Nepalese group consumed more “Cereals, cereal products and substitutes” (897.9 g/day vs. 408.5 g/day, W = 4.510, *p* = 0.010, d_unb_ = 2.749), “Pulses, fresh and processed” (213.0 g/day vs. 28.1 g/day, t(9) = 8.505, *p* < 0.001, d_unb_ = 4.709), “Potatoes, tubers and their products” (395.8 g/day vs. 174.7 g/day, t(9) = 6.675, *p* < 0.001, d_unb_ = 3.700).

In the analysis of individual food groups, differences in consumption of sub-categories also emerged. Within the “Cereals, cereal products and substitutes” food category, the Nepalese consumed more “Rice” than the Italians (838.5 g/day vs. 100.0 g/day, U = 0, *p* = 0.007, d_unb_ = 6.970) but the latter, unlike the Nepalese, also ate “Bread”, “Wheat and wheatflour”, “Breakfast cereals” and “Biscuits” (165.0 g/day, 53.3 g/day, 6.7 g/day, 17.1 g/day, respectively). Within “Vegetables, fresh and processed” food category, higher consumption of “tomatoes, fresh” by Nepalese group (123.1/day vs. 13.3 g/day, t(9) = 9.096, *p* < 0.001, d_unb_ = 5.036) compared to the other sub-categories of this category has emerged, while in the Italian group a distribution of consumption emerged among all the sub-categories. Within “Milk, milk products and substitutes” only Italian subjects consumed “Cheese and substitutes” and the “Milk, milk-based beverages” consumption resulted higher than the Nepalese one (240.7 g/day vs. 5.6 g/day, U = 0, *p* = 0.005, d_unb_ = 2.514). Within “Water and other non-alcoholic beverages” category, Nepalese consumed more “Tap water as such, in beverages or recipes” (1673.3 g/day vs. 1429.7 g/day, t(9) = 1.114, *p* = 0.296, d_unb_ = 0.616), while the Italians drank more “Coffee, tea, herbal tea and substitutes” (653.7 g/day vs. 420.0 g/day, t(9) = 2.495, *p* = 0.034, d_unb_ = 1.381).

Table 3 reports the intakes of EI and nutrients (including supplements) among the two group under study. Mean energy intake was similar. Mean intake of fat was greater in Italians (W = 3.539, *p* = 0.021, d_unb_ = 2.149). The mean contribution of fat and PUFA to total EI was greater in Italians (t(9) = 9.223, *p* < 0.001, d_unb_ = 5.106, and W = 6.600, *p* = 0.001, d_unb_ = 3.967, respectively). Mean contribution of protein intake in grams per kilogram of body weight was very similar among the two groups. Available carbohydrates contribution on food energy was greater in Nepalese (t(9) = 10.102, *p* < 0.001, d_unb_ = 5.593). To be specific, the diet of Italians was found to have a higher density of simple carbohydrates than that of Nepalese (t(9) = 6.879, *p* < 0.001, d_unb_ = 3.809).

The diet of Nepalese was found to have a greater density of dietary fibre than that of Italians (t(9) = 4.426, *p* = 0.002, d_unb_ = 2.450). Also dietary fibre normalised per 1000 Kcal of energy intake was greater in Nepalese (t(9) = 11.208, *p* < 0.001, d_unb_ = 6.205). The most evident difference between the two groups was observed for cholesterol intake, much greater in Italians (U = 1, *p* = 0.010, d_unb_ = 2.715). Only Italians consumed alcoholic beverages (U = 6, *p* = 0.045, d_unb_ = 1.502).

The intake of iron and potassium was lower in Italians (t(9) = 4.266, *p* = 0.002, d_unb_ = 2.362, and t(9) = 3.261, *p* = 0.010, d_unb_ = 1.806, respectively). Instead, the intake of calcium was higher in Italians than in Nepalese (W = 3.179, *p* = 0.029, d_unb_ = 1.925). Mean intakes were found to decrease from Italians to Nepalese group for vitamin A (retinol equivalents: t(9) = 5.771, *p* < 0.001, d_unb_ = 3.195, and retinol: U = 0, *p* = 0.005, d_unb_ = 3.205), riboflavin (U = 0, *p* = 0.004, d_unb_ = 3.567), vitamin K (U = 0, *p* = 0.004, d_unb_ = 3.107), vitamin E (t(9) = 2.848, *p* = 0.019, d_unb_ = 1.577), vitamin D (U = 0, *p* = 0.005, d_unb_ = 3.043) and B12 (U = 0, *p* = 0.004, d_unb_ = 3.107). Instead, mean intakes were found to decrease from Nepalese to Italian group for thiamine (t(9) = 2.601, *p* = 0.029, d_unb_ = 1.440) and vitamin B6 (t(9) = 4.218, *p* = 0.002, d_unb_ = 2.335). The intake of linoleic acid was higher in Italians than in Nepalese (W = 3.519, *p* = 0.020, d_unb_ = 2.123), as well as the intake of linolenic acid (W = 4.372, *p* = 0.010, d_unb_ = 2.650). No other relevant results were found.

Comparing the results with the guidelines, the level of EI was found to be low in both groups for the kind of activity performed (2793 kcal/day of Italian group and 2775 kcal/day of Nepalese group vs. 2000–7000 kcal/day recommended), as well as intake of water (3099 ± 462 g/day of Italian group and 3240 ± 310 g/day of Nepalese group vs. 4000–5000 g/day recommended), intake of protein (1.2 ± 0.4 g/kg bw/day of Italian group and 1.3 ± 0.3 g/kg bw/day of Nepalese group vs. 1.2–2.0 g/kg bw/day recommended), intake of available carbohydrate intake (5 ± 2 g/kg bw/day of Italian group and 7 ± 2 g/kg bw/day of Nepalese group vs. 8–12 g/kg bw/day recommended).

Regarding micronutrients and cholesterol, the intakes were compared to the recommended daily allowances for the Italian population (SINU), not only for Italians but also for Nepalese. A higher intake than recommended levels emerged only in the Italian group for cholesterol (510 ± 190 mg/day vs. max 300 mg/day recommended). From this comparison also emerged that, in both groups, the intake of some micronutrients was lower than the recommended levels, such as calcium and vitamin D. While the intake was higher for phosphorus, magnesium, iron, zinc, thiamine, niacin, vitamin C, vitamin B6, vitamin E. Intake higher than the levels recommended in the Italians, but lower in the Nepalese, was found for vitamin k, vitamin A and vitamin B12.

### 3.2. Anthropometrics and BIA

**Figure 2:**
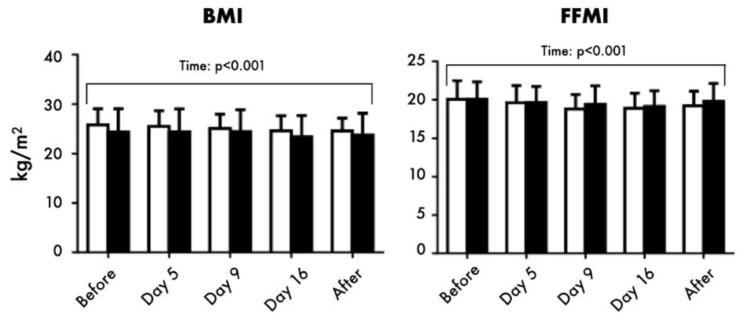
Body mass index (BMI) and Fat-Free Mass Index (FFMI) of participants, before, during (day 5, 9 and 16) and after the altitude expedition. White columns refer to the Italian group, and black columns to the Nepalese group. Data are shown as mean and standard deviation.

Regarding the anthropometric parameters, the BMI decreased in both groups during the trek (*p* < 0.001, η^2^_p_ = 0.724; ω^2^_p_ = 0.692), particularly at day 15 and after the expedition in respect to baseline (see Figure 2). On the same vein, waist circumference decreased in both groups (*p* = 0.002, η^2^_p_ = 0.336; ω^2^_p_ = 0.265), particularly in Italians (time × ethnicity, *p* = 0.020, η^2^_p_ = 0.248; ω^2^_p_ = 0.170) and with significant reductions from day 9.

**Figure 3:**
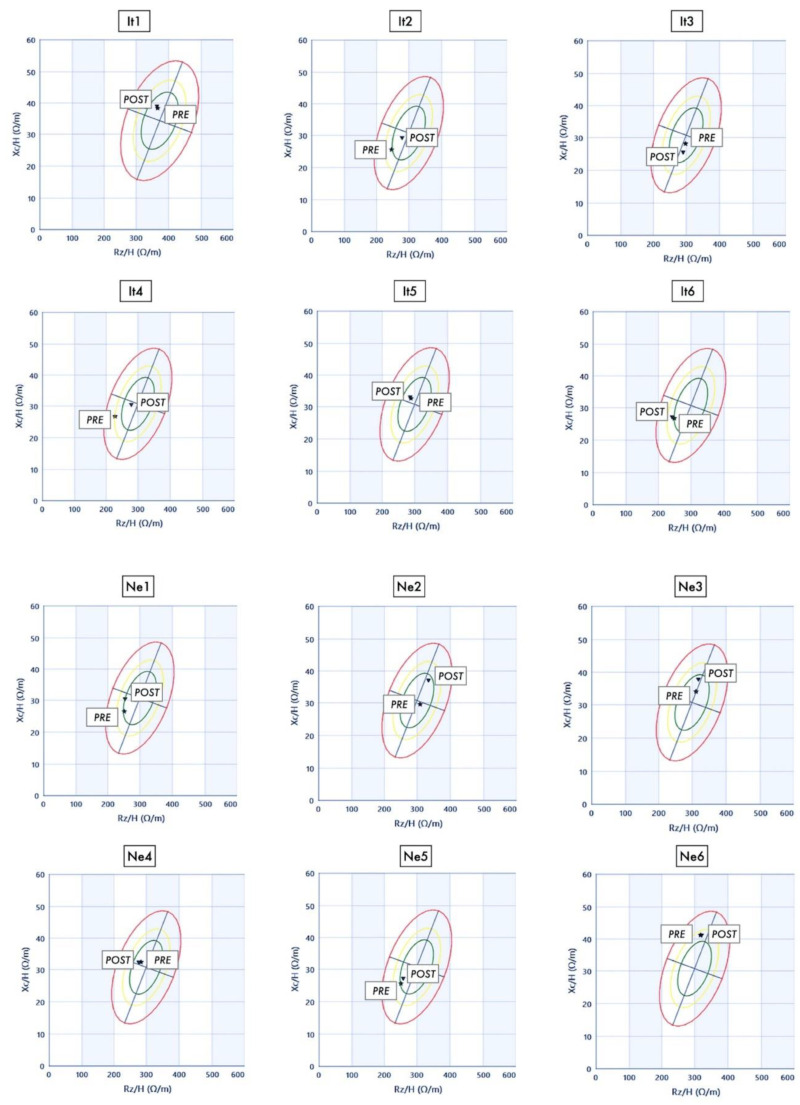
Bioimpedance vector analysis (BIVA) graphs of both Italian (It1–6) and Nepalese (Ne1–6) participants. Raw bioimpedance parameters (i.e., resistance, Rz, and reactance, Xc) are standardised for height and plotted as a vector, of which the direction and length are determined by the hydration status and dielectric mass of soft tissues. The graphs are built according to the up-to-date tolerance ellipses of a reference population (50%, 75% and 95%, from the innermost to the outermost). Stars (PRE) and triangles (POST) represent the first and the last measurement, conducted the day before and the day after the altitude expedition, respectively.

Figure 3 shows the graphs with the BIA vectors of all participants before and after the expedition. The Rz significantly changed during the trek (*p* < 0.001, η^2^_p_ = 0.513; ω^2^_p_ = 0.459) in both groups, with an increase from day 5 to day 9 and 16 with respect to the baseline; these changes were present in both groups. To be considered that Rz increases as the percentage of fat mass increases. The Xc also changed (*p* < 0.001, η^2^_p_ = 0.424; ω^2^_p_ = 0.361) with a significant ethnic difference in the time course (time × ethnicity, *p* = 0.022, η^2^_p_ = 0.243; ω^2^_p_ = 0.165): Italians increased only at day 9, whereas the increment remained significant in Nepalese at day 5, 9, and 16. A similar trend was found for PA (time: *p* = 0.019, η^2^_p_ = 0.275; ω^2^_p_ = 0.198, time × ethnicity, *p* = 0.026, η^2^_p_ = 0.258; ω^2^_p_ = 0.181): a significant increase was found only for Nepalese at day 9 and 16. To be considered that PA increases as body cellular mass increases. Table 4 shows mean values of the waist-to-height ratios and non-estimated BIA values (Rz, Xc, and PhA).

The BMR and BCM parallelly changed during the trek (*p* = 0.042, η^2^_p_ = 0.233; ω^2^_p_ = 0.153 and *p* = 0.038, η^2^_p_ = 0.237; ω^2^_p_ = 0.158, respectively), even if a significant reduction was found only in Italians at day 9. The FFM (see Figure 3) and TBW significantly decreased in response to altitude exposure (*p* < 0.001, η^2^_p_ = 0.478; ω^2^_p_ = 0.421 and *p* < 0.001, η^2^_p_ = 0.476; ω^2^_p_ = 0.418, respectively), with parallel significantly lower values at day 9, 16, and after the expedition in Italians and at day 16 in Nepalese, in respect to baseline.

### 3.3. Muscle Ultrosound

Calculated intraclass correlation coefficient (ICC) indicated an excellent level of reliability for all of the measured parameters (ranging from 0.91–0.98). The analysis of ultrasound images (see Table 5) performed on Italians revealed the CSA of VL was reduced after the expedition (t(5) = 3.321, *p* = 0.021, d_unb_ = 1.142). Instead, no significant difference was found for MT, FL or PA. The CSA of LM did not change significantly, neither in the left nor in the right side.

## 4. Discussion

It is well-documented that above 5000 m weight loss is a major problem for which many explanations have been proposed, including anorexia, elevated basal metabolic rate, body water loss, and altered satiety hormones [10]. Moreover, altitude-induced weight loss is largely due to negative energy balance with suboptimal energy intakes of 50–70% of daily requirements [36,37,38,39,40] and insufficient high-quality protein intake limiting the body’s ability to synthesize protein [10].

The amount of weight loss during mountain travelling is dependent on the amount of duration and altitude exposure [15]. It has been recently reported that a reduction of body weight, body fat and waist circumference occurs after a prolonged sojourn (22 days) at high altitude [16]. The possible beneficial anthropometric adaptations after a period of physical activity at altitude may be related to other beneficial effects on metabolism [41]. Our results agree with these findings, even with a shorter duration and across moderate and high altitude, as the physical strain at altitude produced a body mass reduction in our participants, particularly after 15 days. Waist circumference also decreased in both groups, but to a greater degree in Italians. Our results showed that between the two groups the energy intake was found to be at the lowest level for the physical activity at high altitude even if the intake of macronutrients (as EI%) is almost in line with the nutritional guidelines for moderate/high-intensity training.

At altitude, an increased energy intake may be required for supporting training [42], both due to the effect of a microenvironment with lower temperatures and hard slopes. Therefore, as altitude increases the surplus of energy requirements is more willingly required [8]. On the same vein, the environmental conditions associated with altitude environment elicit special attention to be focused on fluid requirements; therefore, athletes and expeditions groups should consider an increment in water intake and an ongoing evaluation of their hydration status [8]. A balance of fluids and hydration status plays an important role as hypohydration may affect physical function, cognitive performance and health status. The increase in Rz, along with the reduction of TBW, as revealed by BIA, posed the evidence for supporting a special focus on these parameters to continuous monitoring during highly demanding expeditions.

Regarding the other parameters of BIA, PA is generally related to cell integrity, since lowering PA indicates muscle loss; it provides important information on the health and the condition of the subjects. The fact that it increased with a higher degree in Nepalese suggests for these subjects a diverse adaptation to the altitude trekking. However, the increase found in the present study was interpreted not as a muscle mass increment because of the massive changes in body fluids. Indeed, PA can be considered as an indicator of the intra/extra-cellular water content ratio, allowing the interpretation of soft tissue changes in relation to the total water content [19]. BCM is considered a valid index of skeletal muscle mass [43] and it decreased at middle points across the trek. This reduction was parallel to that one of BMR since this last parameter is positively related to the number of body cells. However, the trend of the BCM suggests a high dependence of body fluids changes during the expedition again. The same reason may lie behind the disentanglement of PA and BCM trends.

An appropriate nutrition strategy and adequate hydration are very important to maintain a high and varied nutrient intake in order to avoid changes in body composition [9]. Though, according to many studies performed in the past, it is very difficult to find the right nutritional design. Some authors found that neither additional calories nor higher initial body fat mass attenuated the loss of FFM during a 21-day Himalayan trek [43] and that, despite palatable foods, food intake decreased dramatically, and weight (and FFM) loss occurred [39]. In contrast, others report that it was able to mitigate weight loss by providing food matched precisely to energy expenditure [44] or limiting exercise to minimal tasks and providing palatable food in a comfortable environment [45].

Muscle mass was decreased after the expedition, as revealed by the FFM estimation by BIA, and by the muscle ultrasound. In particular, the ultrasound analysis showed a significant reduction for the CSA (neither thickness, fiber length nor pennation angle) of *Vastus lateralis*, but not of the *Lumbar multifidus*. These findings suggest using cross-sectional area measures, rather than muscle thickness, for monitoring longitudinally skeletal muscle mass adaptation to altitude exposure. In addition, further evidence is needed for demonstrating whether the muscle groups more susceptible to detrimental adaptations during altitude exposure are those more affected by physical strain. To be noted that body fluids alterations are transient, as they reversed immediately after returning at low altitude. In contrast, the loss of muscle mass was a more durable adaptation, and further studies should consider the time course of the adaptation during the follow-up.

A high-protein diet has been suggested at altitude to counteract detrimental body composition changes [16]. In our study, protein intake in terms of grams per kg/body weight was adequate, even though at the lowest level of the recommendations for maintaining FFM mass, as for available carbohydrates. A low calorie and macronutrient intake could be due to the loss of appetite, lack of comfort and palatable food which occur at high altitudes, as widely documented [13,14]. Therefore, reduced oxygen availability, as at altitude, may trigger per se the suppression of protein synthesis through the activation of specific factors [46], albeit an adequate intake of dietary protein. Body fluids balance is regulated, among other systems, through aquaporins [47]. Hypoxia-inducible factors, such as HIF-1, may also regulate body fluid balance through aquaporin function [48]. The presence of aquaporins, such as AQP3, on skeletal myofibers’ membrane [49], and the inducibility of the same AQP by HIF-1 [48], may support a direct role of oxygen deprivation on regulating the structure of skeletal muscles cells. The results of our study show loss of muscle mass and body fluids occurred despite an adequate intake of proteins and specific recommendation for avoiding dehydration. Therefore, we may evoke the role of hypoxia inducible factors in leading such body adaptations, and we advocate for novel recommendations for water and protein intake while exerting physical training or trekking plan at high altitude.

The different intake levels of some micronutrients that emerged from this study reflect only the different food sources available for the two groups during the trek. In fact, Nepalese with a largely vegetable based-diet (mainly without meat, milk, fish, milk and milk products, eggs) had a lower intake than recommended levels for calcium, riboflavin, and vitamin A, K and B12. On the other hand, Italians with a more wide-ranging diet (with animal and vegetable foods) showed an intake only slightly below the recommendations for potassium, calcium and fibres, and much higher for cholesterol. This could be due to one limitation of this study; namely, diet plans not defined before the expedition, since it was conducted in ecological field design. However, it represented the ecological strength of our design, since the two groups commonly differ in terms of dietary habits. The dietary pattern of the Italians followed the typical Mediterranean diet, even though the Italian diet of the last decades contains much more fats and animal-derived foods, namely meat products, milk products, and eggs. Nepalese cuisine, instead, takes the bases on the Indian and Tibetan cuisine; despite a great variability depending on the diverse regions, people of Nepal consume a lot of rice, with the typical dal bath (above described), in addition to momos and chapati bread. Another limitation, due to logistics, of this study was the absence of US data on Nepalese. Further studies providing quantitative outcomes of muscle morphology in both groups should link the quality of protein sources and the muscle adaptations.

Prospectively, as for expeditions to extreme altitude concerns, food technology has progressed in the intervening 50 years to the point that packaged rations can be adequately fortified with vitamins and minerals that are stable for prolonged periods [50]. Pugh’s recommendation was probably prudent advice in 1953, and multivitamin supplementation is still good ‘‘insurance’’ against suboptimal or sporadic food intake on expeditions today. Easy-to-prepare food that does not require a great deal of thought or preparation [50] and ready-to-eat food are essential at high altitude, however a critical survey of the literature does not appear to be available.

## 5. Conclusions

A compromise between the two types of diet followed by the two groups of this study could be a viable solution to try to avoid important changes in body composition for people who are moving to high altitudes for sport or recreation. Altitude trek elicits transient body fluid reductions in both Western travellers and Himalayan porters, even if with a different time course of adaptation between groups. A reduction in muscle mass also occurred, despite an adequate, albeit relatively low, protein intake.

This is the first study analyzing nutrition intake and body composition during a high- altitude expedition in an ecological study design (i.e., conducted in a real setting, studying the relation of living organisms to one another and the environmental conditions). In fact, in this study, any contextual factors have not been modified, leaving the groups to behave as they wanted during the expedition. The group of expeditioners composed by Western travelers and local porters, eating differently, is indeed a typical scenario of Himalayan expeditions. In addition to medical advice for altitude travelers and recent advancement in the evidence of medical problems of local porters, it is necessary to advocate for nutritional and food-related risk factors advice for the diverse altitude expedition groups, i.e., sojourners, trekkers, athletes, climbers, guides and porters. Before large samples studies will be conducted and specific recommendations will be provided, authors suggest increasing the intake of proteins from high quality sources, increasing the intake of water, a more wide-ranging diet for local porters, increasing the intake of vitamins and minerals. Authors also advocate for the continuous monitoring of body adaptations during altitude expeditions, to arrange dietary plans accordingly.

## Figures and Tables

**Table 1:** Distribution of energy, water and macronutrients intake (%) among the principal meals and snacks in Italian (Ita) and Nepalese (Nep) group, averaged from food consumption diaries of participants.

	Breakfast		Lunch		Dinner		Snacks	
	Ita	Nep	Ita	Nep	Ita	Nep	Ita	Nep
Energy Intake	25%	12%	31%	41%	34%	43%	10%	4%
Water	11%	14%	25%	30%	27%	30%	37%	26%
Protein	28%	8%	26%	44%	35%	47%	11%	1%
Fat	18%	20%	34%	39%	36%	41%	12%	0%
Carbohydrates	31%	11%	30%	41%	31%	43%	8%	5%

**Table 2:** Mean and standard deviation of individual daily consumption by food categories (g/day) and sub-categories (g/day) in the Italian (3 days average) and Nepalese (2 days average) group, and the number of consumers only.

		Italian Group (*n* = 5)			Nepalese Group (*n* = 6)		
Food Categories	Food Sub-Categories	*n* Consumers Only	Mean TOTAL Group	SD *	*n* Consumers Only	Mean TOTAL Group	SD *
*Cereals, cereal products and substitutes*		5	408.5	239.6	6	897.9	42.0
	Bread	5	165.0	94.1	0	0.0	0.0
	Pasta and pasta substitutes	5	65.5	18.2	6	55.0	12.2
	Rice	3	100.0	145.8	6	838.5	41.5
	Wheat and wheat flour	5	53.2	28.6	1	4.4	0.0
	Breakfast cereals	2	6.7	0.0	0	0.0	0.0
	Biscuits	2	17.1	0.0	0	0.0	0.0
	Sweets and sweet snacks without creams, jams and without chocolate toppings	1	1.1	0.0	0	0.0	0.0
*Pulses, fresh and processed*		5	28.1	40.8	6	212.9	31.4
	Pulses, fresh and preserved	5	13.2	9.0	0	0.0	0.0
	Pulses, dried	1	14.9	0.0	6	212.9	31.4
*Vegetables, fresh and processed*		5	247.6	85.7	6	185.7	21.8
	Leafy vegetables, fresh	5	91.7	40.6	0	0.0	0.0
	Tomatoes, fresh	3	13.3	25.9	6	123.1	18.2
	Tomatoes, processed	5	20.0	0.0	0	0.0	0.0
	Other fruiting vegetables, fresh	5	40.0	10.5	0	0.0	0.0
	Roots and onions, fresh	5	28.0	13.1	6	43.9	0.9
	Other vegetables, fresh	5	52.0	31.6	6	18.7	2.8
	Other vegetables, preserved	1	1.9	0.0	0	0.0	0.0
	Spices and herbs	5	0.8	0.3	1	0.02	0.0
*Potatoes, tubers and their products*		5	174.7	39.1	6	395.8	64.5
	Potatoes, potatoes-based products, excluding french fries	5	174.7	39.1	6	395.8	64.5
*Fruit, fresh and processed*		5	117.3	29.1	4	16.7	0.0
	Exoticf ruit, fresh	5	81.7	22.4	4	16.7	0.0
	Other fruit, fresh	4	20.0	0.0	0	0.0	0.0
	Nuts, seeds, olives and their products, dried fruit	5	15.7	6.8	0	0.0	0.0
*Meat, meat products and substitutes*		5	86.9	16.6	0	0.0	0.0
	Beef and veal, not preserved, excl. offal	5	30.9	15.1	0	0.0	0.0
	Pork, not preserved, excl. offal	5	15.6	6.1	0	0.0	0.0
	Poultry and game, not preserved, excl. offal	5	26.7	3.7	0	0.0	0.0
	Other meats, not preserved, excl. offal	2	4.2	5.9	0	0.0	0.0
	Ham, salami, sausages and other preserved meats, excl. offal	3	9.6	4.6	0	0.0	0.0
*Milk, milk products and substitutes*		5	251.4	133.8	1	5.6	0.0
	Milk, milk-based beverages	5	240.7	127.4	1	5.6	0.0
	Cheese and substitutes	3	10.7	9.2	0	0.0	0.0
*Eggs*		5	108.9	49.4	1	11.8	0.0
	Eggs	5	108.9	49.4	1	11.8	0.0
*Oils and fats*		5	71.9	19.7	6	65.1	5.4
	Olive oil	5	17.6	7.3	6	33.5	3.9
	Other vegetable oils	5	53.1	15.5	6	30.5	4.9
	Butter and creams	3	1.3	0.6	1	1.1	0.0
*Sweet products and substitutes*		5	38.7	18.1	6	25.1	5.3
	Chocolate and substitutes	1	1.3	0.0	0	0.0	0.0
	Candies, jam and other sweet products incl. sugar-free	3	4.7	5.1	0	0.0	0.0
	Sugar, fructose, honey and other nutritious sweeteners	5	32.6	17.9	6	25.1	5.3
*Water and other non-alcoholic beverages*		5	2083.8	312.8	6	2093.3	276.8
	Tap water as such, in beverages or recipes	5	1429.7	322.0	6	1673.3	390.1
	Coffee, tea, herbal tea and substitutes	5	653.7	166.9	6	420.0	144.3
	Fruit and vegetable juices	1	0.4	0.0	0	0.0	0.0
*Alcoholic beverages and substitutes*		3	66.0	0.0	0	0.0	0.0
	Beer, cider and substitutes	3	66.0	0.0	0	0.0	0.0
*Meal substitutes*		1	1.8	0.0	0	0.0	0.0
	Meal substitutes	1	1.8	0.0	0	0.0	0.0
*Miscellaneous*		5	3.7	3.1	1	0.2	0.0
	Non-fat sauces or condiments (ketchup, vinegar, etc.)	5	2.9	2.6	0	0.0	0.0
	Nuts for broth or other various products	4	0.8	0.5	1	0.2	0.0
*Food supplements*		3	2.7	2.9	0	0.0	0.0
	Food supplements	3	2.7	2.9	0	0.0	0.0
Total amount of foods and beverages			3692			3910	
Total amount of solid foods			1302			1811	
Total amount of liquid foods **			2391			2099	

* Standard Deviation. ** All beverages and milk in liquid state were classified as liquid foods; all other items were classified as solid foods.

**Table 3:** Mean daily energy and nutrient intakes for Italian and Nepalese group, expressed as mean ± SD, % of Energy Intake, and nutrition guidelines for moderate/high-intensity training (tra GL ^1,2,3^) and Recommended daily allowances of nutrient intake for the generic population (It RDA ^4^).

	Nepalese (*n* = 6)		Italians (*n* = 5)		Tr GL ^1,2,3^	It RDA ^4^
	Mean	SD *	Mean	SD *		
Energy Intake_EI (MJ)	12	3	12	1		
Energy Intake_EI (kcal)	2793	810	2755	188	2000–7000	
Water (g/day)	3099	462	3240	310	4000–5000 ^2^	2500 ^AI^
Protein (g/day)	90	30	84	7		63 ^PRI^
Protein (g/kg/day)	1.2	0.4	1.3	0.3	1.2→2.0 ^1,2^	0.9 ^PRI^
Fat (g/day)	118	27	74	6		
SFA (g/day)	25	6	11	2		
MUFA(g/day)	46	11	37	3		
PUFA (g/day)	39	9	23	3		
Cholesterol (mg/day)	510	190	47	116		<300 ^SDT^
Available carbohydrate (g/day)	362	127	466	33		
Available carbohydrate (g/kg bw/day)	5	2	7	2	8 ^1^–12 ^2^	
Simple carbohydrate (g)	90	15	45	6		
Starch (g/day)	248	116	383	28		
Dietary fibre (g/day)	23	9	43	6		>25 ^SDT^
Dietary fibre (g/1000 Kcal/d)	8.1	0.9	15.6	1.3		12.6–16.7 ^RI^
Alcohol (g/day)	2	2	0.0	0.0		
*% Total energy from*						
Protein (%EI)	13	4	12	1		
Fat (%EI)	38	9	24	5	up to 50 ^1^	≤30 ^RI^
SFA (%EI)	8	2	4	1	pRDA °	<10 ^SDT^
PUFA (%EI)	12	3	7	1	pRDA °	5–10 ^RI^
Available carbohydrate (%EI)	49	17	63	5		45–60 ^RI^
Simple carbohydrate (%EI)	13	4	6	1		<15 ^SDT^
*Minerals*						
Potassium (mg/day)	3815	993	5479	700	pRDA °	3900 ^AI^
Phosphorus (mg/day)	1446.4	449.6	1453.0	129.6	pRDA °	700 ^PRI^
Calcium (mg/day)	789	219	468	57	pRDA °	1000 ^PRI^
Magnesium (mg/day)	408	72	462	48	pRDA °	240 ^PRI^
Iron (mg/day)	14	5	24	3	Up to 70% of requirement	10 ^PRI^
Zinc (mg/day)	14	4	17	2	pRDA °	12 ^PRI^
*Vitamins*						
Thiamine (mg/day)	1.3	0.4	1.8	0.2	pRDA °	1.2 ^PRI^
Riboflavin (mg/day)	1.9	0.4	1.0	0.1	pRDA °	1.6 ^PRI^
Niacin (mg/day)	21	4	21	3	pRDA °	18 ^PRI^
Vitamin C (mg/day)	121	22	114	16	pRDA °	105 ^PRI^
Vitamin B6 (mg/day)	3	1	5	1	pRDA °	1.3 ^PRI^
Vitamin A (REs µg/day) ^§^	855	228	284	81	pRDA °	700 ^PRI^
Vitamin K (µg /day)	831	344	52	6	pRDA °	140 ^AI^
Vitamin E (mg/day)	43	11	30	4	pRDA °	13 ^AI^
Vitamin D (µg/day)	3.1	1.2	0.2	0.5	pRDA °	15 ^AI^
Vitamin B12 (µg/day)	3.7	1.0	0.2	0.4	pRDA °	2.4 ^PRI^

* Standard Deviation; ° Recommended daily allowances of nutrient intake for the general population (SINU); ^§^ Vitamin A expressed as retinol equivalents (REs): 1 retinol equivalent (RE) = 1 µg retinol = 6 µg β-carotene; AI—Adequate Intake; AR—Average Requirement; PRI—Population Recommended Intake; RI—Reference intake range for nutrients; SDT—Suggested Dietary Targets; ^1^ Kerksick et al. 2018 [25]; ^2^ Thomas et al. 2016 [26]; ^3^ Carlsohn et al., 2016 [27]. ^4^ *SINU*, 2014 [35].

**Table 4:** Mean values of anthropometric tests and bioimpedance analysis during the expedition in both group of participants.

	Before		Day 5		Day9		Day 16		After	
	Ita	Nep	Ita	Nep	Ita	Nep	Ita	Nep	Ita	Nep
WtHR	0.53	0.51	0.52	0.50	0.51	0.49	0.50	0.50	0.50	0.50
Rz/h (Ω)	482.80	476.00	497.30	508.30	530.00	515.50	520.20	511.20	501.70	486.80
Xc/h (Ω)	51.67	52.50	52.50	63.83	57.67	60.17	57.00	61.67	53.50	57.33
PhA (degree)	6.13	6.28	6.03	7.13	6.25	6.63	6.27	6.88	6.08	6.70

WtHR: waist-to-height ratio; Rz: resistance; Xc: capacitive reactance; PhA: phase angle.

**Table 5:** Measured muscle ultrasound parameters at baseline and after the altitude expedition on Italians.

	Before		After		
	Mean	SD	Mean	SD	95% Confidence Interval
CSA, VL (cm^2^) *	16.78	4.05	15.57	3.41	−0.27 to −2.15
MT, VL (cm)	2.17	0.38	2.13	0.35	0.10 to −0.18
FL, VL (cm)	7.40	0.61	7.50	0.57	0.35 to −0.15
PA, VL (°)	15.51	0.87	14.95	1.50	0.59 to −1.71
CSA, r-LM (cm^2^)	13.13	1.90	12.49	1.43	0.61 to −2.15
CSA, l-LM (cm^2^)	13.15	1.96	12.38	1.54	0.56 to −1.85

SD: standard deviation; CSA: cross-sectional area; VL: *Vastus lateralis*; MT: muscle thickness; FL: fibre length; PA: pennation angle; l: left side; r: right side; LM: *Lumbar multifidus*. 95% CI refers to the mean difference; * *p* < 0.05.

## Data Availability

The data presented in this study are available on request from the corresponding author.
